# Analysis of Side Effects Following Vaccination Against COVID-19 Among Individuals With Multiple Sclerosis Treated With DMTs in Poland

**DOI:** 10.3389/fneur.2022.913283

**Published:** 2022-06-14

**Authors:** Agata Czarnowska, Joanna Tarasiuk, Olga Zajkowska, Marcin Wnuk, Monika Marona, Klaudia Nowak, Agnieszka Słowik, Anna Jamroz-Wiśniewska, Konrad Rejdak, Beata Lech, Małgorzata Popiel, Iwona Rościszewska-Żukowska, Adam Perenc, Halina Bartosik-Psujek, Mariola Świderek-Matysiak, Małgorzata Siger, Agnieszka Ciach, Agata Walczak, Anna Jurewicz, Mariusz Stasiołek, Karolina Kania, Klara Dyczkowska, Alicja Kalinowska-Łyszczarz, Weronika Galus, Anna Walawska-Hrycek, Ewa Krzystanek, Justyna Chojdak-Łukasiewicz, Jakub Ubysz, Anna Pokryszko-Dragan, Katarzyna Kapica-Topczewska, Monika Chorąży, Marcin Bazylewicz, Anna Mirończuk, Joanna Kulikowska, Jan Kochanowicz, Marta Białek, Małgorzata Stolarz, Katarzyna Kubicka-Bączyk, Natalia Niedziela, Paweł Warmus, Monika Adamczyk-Sowa, Aleksandra Podlecka-Piçtowska, Monika Nojszewska, Beata Zakrzewska-Pniewska, Elżbieta Jasińska, Jacek Zaborski, Marta Milewska-Jȩdrzejczak, Jacek Zwiernik, Beata Zwiernik, Andrzej Potemkowski, Waldemar Brola, Alina Kułakowska

**Affiliations:** ^1^Department of Neurology, Medical University of Białystok, Białystok, Poland; ^2^Faculty of Economic Sciences, University of Warsaw, Warsaw, Poland; ^3^Department of Neurology, Jagiellonian University Medical College, Krakow, Poland; ^4^Department of Neurology, Medical University of Lublin, Lublin, Poland; ^5^Neurology Clinic With Brain Stroke Sub-Unit, Clinical Hospital No. 2 in Rzeszow, Lwowska, Poland; ^6^Department of Neurology, Institute of Medical Sciences, Medical College of Rzeszow University, Rzeszów, Poland; ^7^Department of Neurology, Medical University of Lodz, Łódz, Poland; ^8^Department of Neurology, Poznan University of Medical Sciences, Poznań, Poland; ^9^Division of Neurochemistry and Neuropathology, Poznan University of Medical Sciences, Poznań, Poland; ^10^Department of Neurology, Faculty of Medical Sciences in Katowice, Medical University of Silesia, Katowice, Poland; ^11^Department of Neurology, Wroclaw Medical University, Wroclaw, Poland; ^12^Department of Neurology, Regional Specialised Hospital No. 4 in Bytom, Bytom, Poland; ^13^Department of Neurology, Faculty of Medical Sciences in Zabrze, Medical University of Silesia in Katowice, Katowice, Poland; ^14^Department of Neurology, Medical University of Warsaw, Warsaw, Poland; ^15^Jan Kochanowski University, Collegium Medicum, Kielce, Poland; ^16^Clinical Center, Resmedica, Kielce, Poland; ^17^Department of Neurology and Neurorehabilitation, Miedzyleski Szpital Specjalistyczny, Warsaw, Poland; ^18^Department of Neurology and Ischemic Strokes, Medical University of Lodz, Lodz, Poland; ^19^Neurology Ward, Provincial Specialist Hospital, Olsztyn, Poland; ^20^Department of Neurology, University of Warmia and Mazury, Olsztyn, Poland; ^21^Department of Neurology, University of Warmia and Mazury, Olsztyn, Poland; ^22^Clinic of Neurology, University of Warmia and Mazury, Olsztyn, Poland; ^23^Department of Clinical Psychology and Psychoprophylaxis, University of Szczecin, Szczecin, Poland; ^24^Department of Neurology, Specialist Hospital in Końskie, Collegium Medicum, Jan Kochanowski University in Kielce, Kielce, Poland

**Keywords:** multiple sclerosis, vaccination, SARS-CoV-2, COVID-19, side effects

## Abstract

**Background and Objectives:**

Since vaccination against COVID-19 is available for over a year and the population of immunized individuals with autoimmune disorders is higher than several months before, an evaluation of safety and registered adverse events can be made. We conducted a large study of side effects following the COVID-19 vaccine among patients with multiple (MS) sclerosis treated with disease-modifying therapies (DMTs) and analyzed factors predisposing for particular adverse events.

**Methods:**

We gathered data of individuals with MS treated with DMTs from 19 Polish MS Centers, who reported at least one adverse event following COVID-19 vaccination. The information was obtained by neurologists using a questionnaire. The same questionnaire was used at all MS Centers. To assess the relevance of reported adverse events, we used Fisher's exact test, *t*-test, and *U*-Menn-Whutney test.

**Results:**

A total of 1,668 patients with MS and reports of adverse events after COVID-19 vaccination were finally included in the study. Besides one case marked as “red flag”, all adverse events were classified as mild. Pain at the injection site was the most common adverse event, with a greater frequency after the first dose. Pain at the injection site was significantly more frequent after the first dose among individuals with a lower disability (EDSS ≤2). The reported adverse events following immunization did not differ over sex. According to age, pain at the injection site was more common among individuals between 30 and 40 years old, only after the first vaccination dose. None of the DMTs predisposed for particular side effects.

**Conclusions:**

According to our findings, vaccination against COVID-19 among patients with MS treated with DMTs is safe. Our study can contribute to reducing hesitancy toward vaccination among patients with MS.

## Introduction

The long-term impact of the severe acute respiratory syndrome coronavirus-2 (SARS-CoV-2) infection on individuals with autoimmune disorders is unknown. Among patients with multiple sclerosis (MS), the course of the infection can be severe in those with a higher level of disability, comorbid diseases, older, and on high effective therapies (1).

In general, vaccination is recommended for individuals with MS. Systemic infection can worsen the course of MS, so prevention is advisable. Most vaccines are considered safe for patients under disease-modifying therapies (DMTs). However, live vaccines are contraindicated under immunosuppressive treatment in most cases (2).

The first vaccines against coronavirus disease 2019 (COVID-19) were approved by the end of 2020. Their high effectiveness was reported in early studies. The mortality and hospitalization rate of SARS-CoV-2 infection is significantly lower in vaccinated persons (3, 4). Throughout the COVID-19 pandemic, diverse variants of SARS-CoV-2 have emerged. The latest variant (Omicron) seems to be more infectious than the original virus (5). The effectiveness of vaccination varies across virus variants and is still under investigation. However, a beneficial role of vaccination is suggested against old and novel variants. The proposed mechanism behind this is the immunological T cell memory induced by vaccination to cross-recognize different variants (6). Therefore, vaccination against COVID-19 is highly recommended, especially for those with autoimmune and other comorbid diseases (7).

Numerous adverse events were reported after the COVID-19 vaccination. However, the overwhelming majority of side effects are mild and self-limiting. In rare cases, serious post-vaccine incidents were observed, including neurological side effects (8).

Here we report adverse events after COVID-19 vaccination among individuals with multiple sclerosis treated with different disease-modifying therapies in Poland and identify any predisposing factors for the occurrence of side effects.

## Materials and Methods

The Multiple Sclerosis and Neuroimmunology Section of the Polish Neurological Society published an announcement about the study at www.ptneuro.pl, and every MS Center in Poland was invited to participate. Finally, participants were recruited from 19 Polish MS Centers. The data was obtained by neurologists using a questionnaire. The same questionnaire was used at all MS Centers (available in Supplementary Materials). Patients were recruited to the study during standard or unplanned visits at a particular MS Center or over the telephone.

We included individuals who had any adverse event after COVID-19 vaccination and confirmed diagnosis of MS according to 2010 and 2017 McDonald criteria. Disability was assessed by the Expanded Disability Status Scale (EDSS). All patients were treated with one of the DMTs available in Poland (interferon, glatiramer acetate, teriflunomide, dimethyl fumarate, fingolimod, alemtuzumab, cladribine, natalizumab, or ocrelizumab).

We collected patient demographics, data regarding specific features of multiple sclerosis, information about vaccination against SARS-CoV-2, presence of adverse events after vaccination), and information regarding relapses following immunization or worsening of MS symptomatology. Incidence classified as relapse must have had a clear monophasic course, objective findings typical of multiple sclerosis verified by a neurologist, lasted over 24 h, and were not related to fever or infection. Gathered data included side effects after the first or second dose of different vaccines. The analysis did not include side effects after the third dose, as the observation time would be insufficient and the number of patients too little.

Categorical variables were characterized by frequency and percentage. Continuous variables were reported by their median, mean value, and interquartile range. For statistical comparisons, the χ2 test of homogeneity of odds was calculated. To assess the relevance of reported adverse events, we used Fisher's exact test, *t*-test, and *U*-Mann-Whitney test.

All calculations were performed using STATA 15 software (StataCorp 2017) (7).

The study was approved (approval No. 62/2021) by the Bioethics Committee at Collegium Medicum, Jan Kochanowski University in Kielce, Poland.

## Results

A total of 1,668 individuals with MS and reports of adverse events after COVID-19 vaccination were included in the study. Among participating MS Centers 3,264 patients were vaccinated with at least one dose. Therefore, the percentage of individuals reporting any adverse events was 51% and the percentage of patients denying any side effects was 49%. Thirty-seven patients with missing data were excluded. Demographic and clinical data regarding features of multiple sclerosis are presented in Table 1. The average observation time was 7 months (range: 1–12 months).

**Table 1:** Demographics and clinical characteristics of patients with MS who presented with side effects following vaccination against SARS-CoV-2.

	**N**	**(%)**	**Mean**	**Median**	**IQR**	**SD**
Study population	1,668	100				
Sex						
Female	1,209	72.48				
Male	459	27.52				
Whole study population age			41.88	42	16	11.07
Female age			42.03	42	16	11.21
Male age			41.49	41	16	10.68
Disease course						
RRMS	1,585	95.02				
SPMS	42	2.52				
PPMS	41	2.46				
EDSS			2.36	2	2.5	1.48
≤2	917	54.98				
3–4	658	39.44				
≥5	93	5.58				
Disease duration			9.44	8	9	6.34
DMTs						
Interferon beta	377	22.6				
Glatiramer acetate	134	8.03				
Dimethyl fumarate	665	39.87				
Teriflunomide	168	10.07				
Fingolimod	74	4.44				
Natalizumab	105	6.29				
Ocrelizumab	77	4.62				
Cladribine	14	0.84				
Alemtuzumab	10	0.6				
Mitoxantrone	6	0.36				
Others	38	2.28				

*AMS, multiple sclerosis; SD, standard deviation; IQR, interquartile range; RRMS, relapsing-remitting multiple sclerosis; PPMS, primary progressive multiple sclerosis; SPMS, secondary progressive multiple sclerosis; EDSS, the expanded disability status scale; DMTs, disease-modifying therapies*.

The distribution of vaccines against SARS-CoV-2 administered among the cohort was as follows: 1,215 (72.84%) patients immunized with the BioNTech-Pfizer vaccine, 223 (13.37%) with the Oxford-Astra Zeneca vaccine, 155 (9.29%) with the Moderna vaccine, and 75 (4.5%) with the Johnson & Johnson vaccine. More than three-quarters (77.34%) of individuals were administered vaccines using genetically engineered mRNA to induce an immune response (BioNTech Pfizer vaccine; Moderna vaccine). The first vaccination dose was given to all patients and the second to 1,573 (94.3%) people.

**Figure 1:**
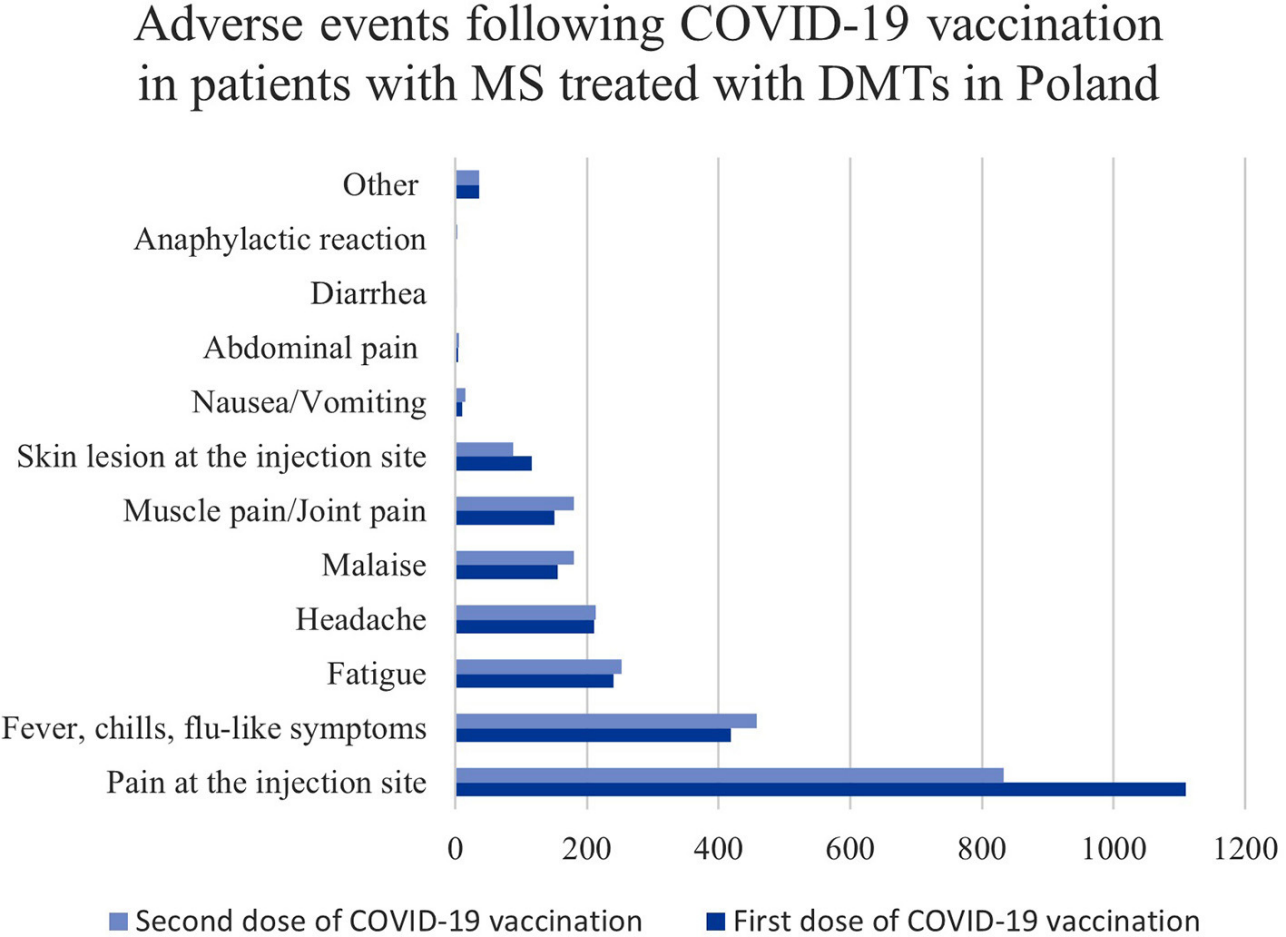
The distribution of adverse events reported in individuals with MS treated with DMTs in Poland.

The reported adverse events were almost exclusively mild. The distribution of particular side effects among the cohort is presented in Figure 1. The most common, with a greater frequency after the first dose, was pain at the injection site. Fever/chills/flu-like symptoms, fatigue, headache, malaise, and muscle/joint pain were more often present after the second dose. In the majority of cases, the reported symptoms were self-limiting. The adverse events resolved within 7 days in 98.3% of patients after the first dose and 97.6% after the second dose. The proportion of most common adverse events following particular vaccines is shown in Table 2. All differences were statistically significant.

**Table 2:** The proportion of most common side effects after particular vaccines administered among the cohort.

	**Pain at the injection site**	**Skin lesion at the injection site**	**Fever, chills, flu-like symptoms**	**Fatigue**	**Headache**	**Muscle pain/Joint pain**	**Malaise**
	**(%)**	**(%)**	**(%)**	**(%)**	**(%)**	**(%)**	**(%)**
**First dose**							
BioNTech, Pfizer vaccine	68.89	4.86	20.08	12.59	10.04	7.16	7.08
Oxford, Astra Zeneca Vaccine	60.99	11.21	43.05	22.42	22.42	17.04	20.18
Moderna vaccine	64.52	15.48	27.74	16.13	16.77	10.97	10.97
Johnson & Johnson vaccine	49.33	10.67	48	17.33	17.33	12	10.67
Pearson chi2	0.001	0.000	0.000	0.001	0.000	0.000	0.000
Fisher's exact *p*-value	0.001	0.000	0.000	0.002	0.000	0.000	0.000
**Second dose**							
BioNTech, Pfizer vaccine	54.98	4.53	26.42	14.73	10.29	9.3	9.71
Oxford, Astra Zeneca Vaccine	36.77	8.97	26.01	16.14	24.66	12.56	10.76
Moderna vaccine	52.26	8.39	50.32	24.52	20	24.52	24.52
Pearson chi2	0.000	0.002	0.000	0.000	0.000	0.000	0.000
Fisher's exact *p*-value	0.000	0.002	0.000	0.000	0.000	0.000	0.000

The mRNA vaccines significantly predisposed for developing pain at the injection site in comparison to vaccines using non-replicating viral vectors (Oxford-Astra Zeneca vaccine; Johnson & Johnson vaccine) (*p* = 0.001). However, being administered with vector vaccines increased propensity for fever, headache, fatigue, skin lesion at the injection site, and muscle/joint pain following immunization (*p* = 0.000, *p* = 0.000, *p* = 0.001, *p* = 0.004; *p* = 0.000, respectively).

**Figure 2:**
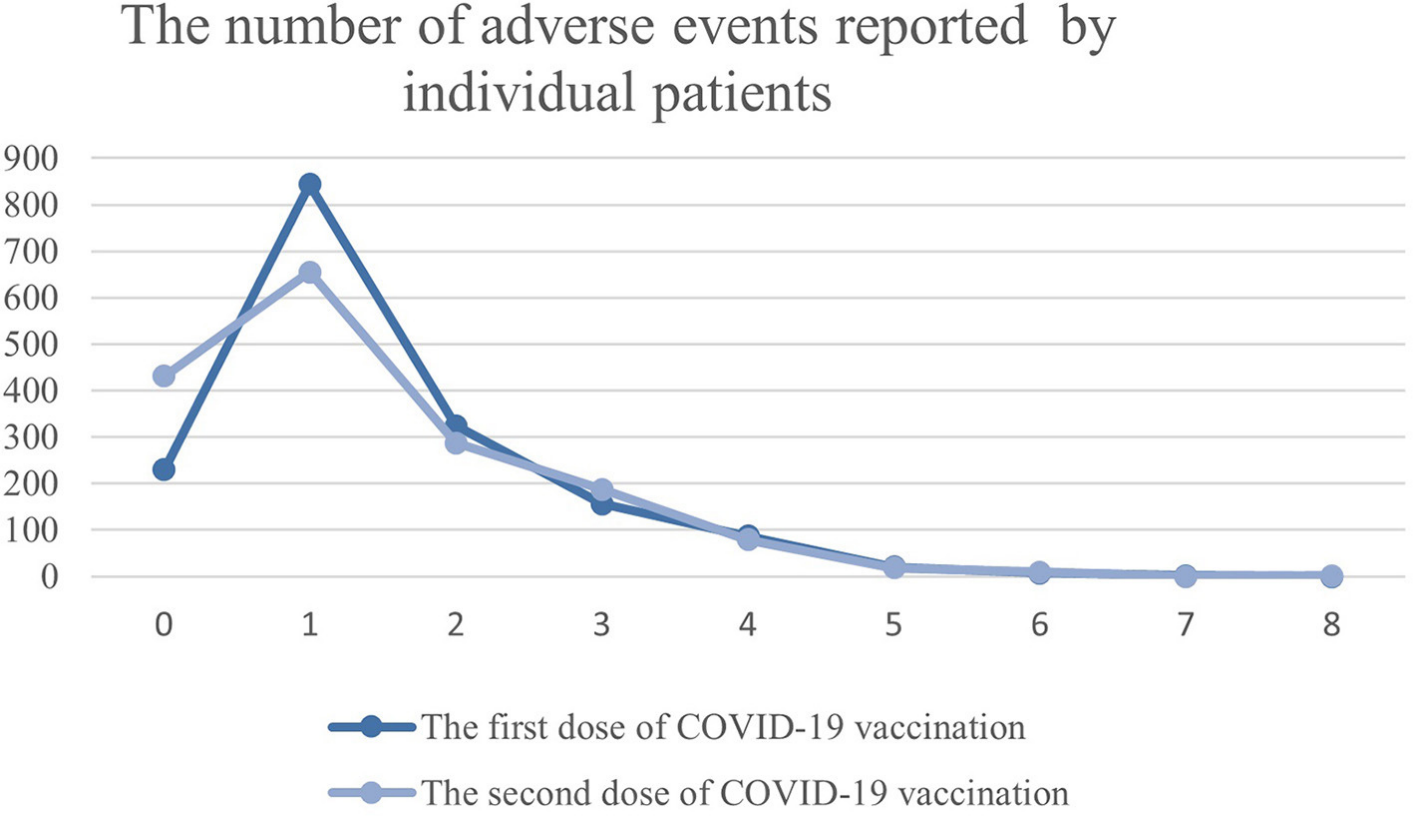
The number of adverse events reported by individual patients with MS treated with DMTs in Poland.

Generally, the observed side effects were not multisymptomatic. After the first dose, 844 (50.6%) individuals had one adverse event and 655 (41.64%) after the second dose. The number of reported adverse events by individual patients is shown in Figure 2.

Only one adverse event was classified as “Red-Flag”. It was a pro-thrombotic incidence in a 42 years old female patient 2 weeks after the first dose of the Oxford-Astra Zeneca vaccine. The patient complained of chest pain, the laboratory finding showed elevated D-dimers level, but pulmonary embolism was excluded. Currently, the patients fells well and further diagnostics did not confirm any thromboembolism. Three patients had anaphylactic reactions immediately after immunization (one individual after both doses). There were no fatal outcomes following vaccination.

None of the DMTs significantly predisposed for particular adverse events or longer duration of side effects. However, the sample size for cladribine, alemtuzumab, and mitoxantrone was insufficient for statistical analysis.

**Figure 3:**
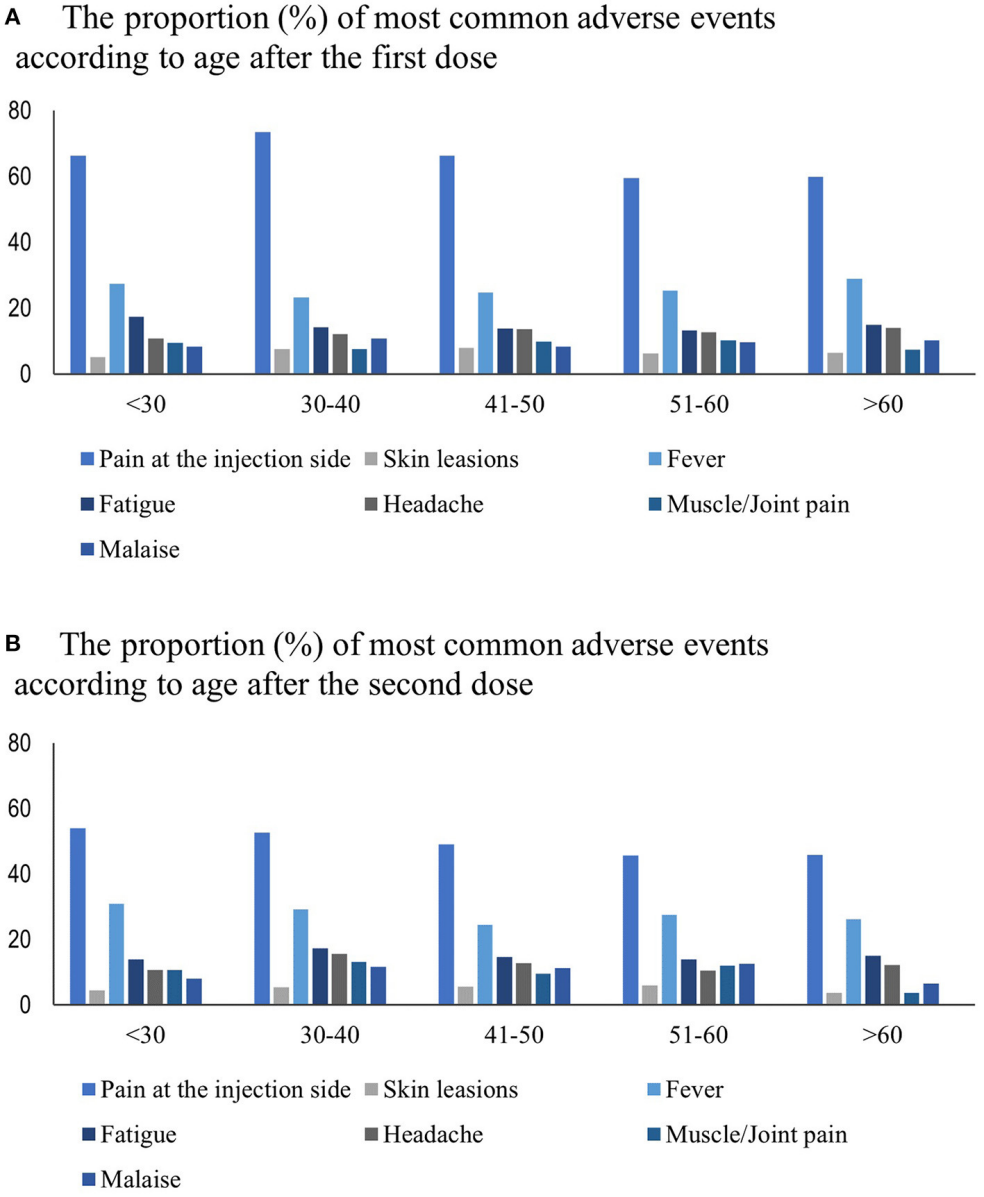
**(A)** The proportion (%) of most common adverse events according to age after the first dose. **(B)** The proportion (%) of most common adverse events according to age after the second dose.

The reported adverse events following immunization did not differ between sex. According to age, pain at the injection site was more common among individuals between 30 and 40 years old, only after the first vaccination dose (*p* = 0.001). The proportion of most common adverse events divided by age is shown in Figures 3A,B. The mean duration of the disease was similar for all side effects, there were none relevant differences.

**Figure 4:**
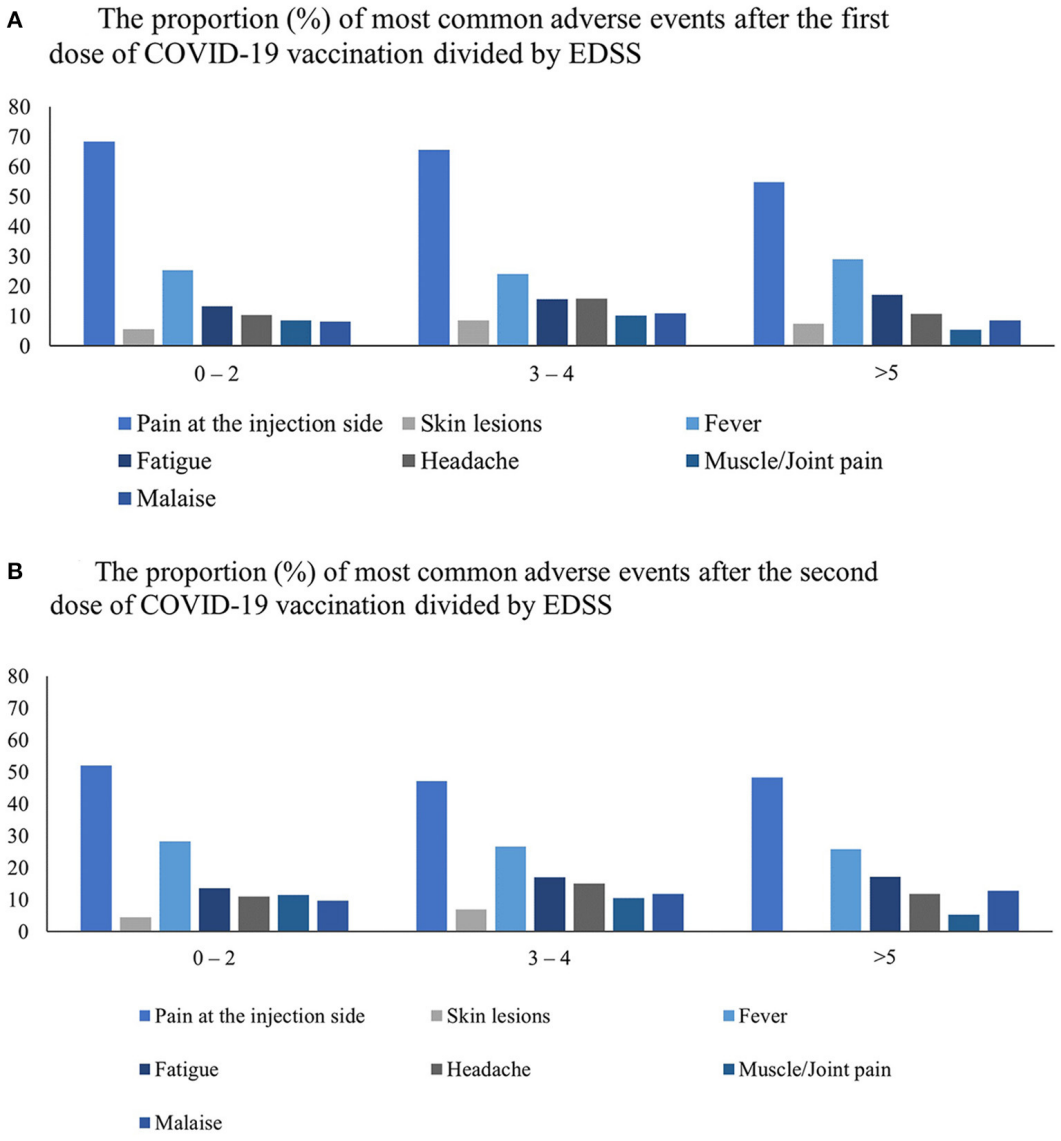
The proportion (%) of most common side effects after COVID-19 vaccination among the cohort was divided by three categories of disability assessed by EDSS after the first **(A)** and the second dose **(B)**.

Pain at the injection site was significantly more frequent after the first dose among individuals with a lower disability (EDSS ≤ 2) (*p* = 0.027). However, a headache was the dominant adverse event after the first dose in individuals with moderate disability (EDSS 3–4) (*p* = 0.005). The proportion of patients with the most common adverse events divided by EDSS is shown in Figures 4A,B.

Among individuals with RRMS, 4.42% of patients (70 people) had relapses up to 3 months before vaccination. After immunization (up to 3 months), 67 patients (4.02%) had relapsed, but only 22.39% of them within the first 21 days. In 29 cases (1.74%), the worsening occurred after the first dose and in 38 (2.28%) after the second dose. Magnetic resonance imaging (MRI) was not routinely performed.

## Discussion

The real-world data regarding vaccination against SARS-CoV-2 among individuals with autoimmune disorders is limited. This study analyzes the range of adverse events following the COVID-19 vaccine reported in patients with multiple sclerosis treated with DMTs.

In our observation, almost all reported adverse events were mild and self-limiting. The most common were pain at the injection site, fever/chills/flu-like symptoms, and fatigue. A similar range and frequency of adverse events were found in clinical trials evaluating COVID-19 vaccinations in general population (9). Pain at the injection site after the first dose of vaccination was more common for individuals with lower disability and patients under 40 years old. The same observations were made on a smaller cohort of people with SM by Achiron et al. (10). In several other studies, based on the general population, also younger patients reported any adverse events more often (11, 12). Therefore, the shift toward younger patients may not be related to the coexistence of autoimmune diseases.

Three patients had developed anaphylactic reactions immediately after immunization. Only one patient had a pro-thrombotic “Red-Flag” (chest pain, elevated D-dimers level) without a final diagnosis of any embolism.

Interestingly, in clinical trials of COVID-19 vaccines, the percentage of adverse events in the placebo group was quite high (approximately one-third). The most frequent were headaches and fatigue (13). It is important to acknowledge the fact, as the mentioned symptoms were also common among patients with MS and, in some cases, might be related to other factors (e.g., anxiety related to the safety of the vaccine).

The occurrence of relapses following vaccination was very low in our cohort and not higher in comparison to the 3 month period before immunization. There are case reports in the literature showing a temporal relation between the COVID-19 vaccine and relapse (14). However, the greater frequency of relapses following vaccination against SARS-CoV-2 has not been observed in our study or other studies conducted on a larger number of patients, including the third dose (10, 14, 15).

None of the DMTs among the cohort were predisposed to a particular adverse event. There was no difference between monoclonal antibodies, sphingosine-1-phosphate receptor modulators, and other therapies in terms of type or the duration of reported side effects. Patients with MS were vaccinated in Poland, keeping a time interval between the administration of certain DMTs according to guidelines, consistent with international consensus (16). The vast majority of our cohort was administered vaccines based on mRNA. Therefore, based on our findings, we can conclude that mRNA vaccines are safe, even on high-efficacy therapies. Among individuals immunized with the use of non-replicating viral vectors, the reported adverse events were also mild, but as the number of patients was much smaller in comparison to patients administered with mRNA vaccines, a larger observation is required to draw conclusions.

The results of our study provide an argument for pro immunization among hesitating individuals. As we know from several studies, there are multiple issues holding patients back from getting vaccinated (17, 18). Most are related to the novelty of the vaccination and concerns about its safety. Also, their effectiveness is constantly undermined by false information on the Internet and social media (19). This creates a big challenge for health workers worldwide. Most clinical trials are based on the general population. Therefore, our study proving vaccination safety among individuals with MS can be a convincing tool for these particular patients.

There are several limitations to our study. Although the study included a large representation of patients with MS treated with DMTs in Poland, the total number of individuals treated with DMTs is much higher. We did not include non-treated with DMTs patients and those with a high level of disability (EDSS >8). Furthermore, the representation of different types of MS is unequal in the cohort as mostly patients with RRMS are included. Finally, the reports of adverse events were in most cases retrospective and based, besides relapses, on subjective assessment of the patient, so it might be imprecise in some individuals.

## Conclusions

The reason for COVID-19 vaccination hesitancy is multifactorial. However, there are genuine fears of potential adverse events, especially among individuals with autoimmune diseases. Our study demonstrates the safety of vaccination against SARS-CoV-2 among patients with MS treated with DMTs. Almost all reported symptoms were mild and self-limiting, some were more frequent in younger patients and with lower EDSS.

## Data Availability Statement

The datasets presented in this study can be found in online repositories. The name of the repository and accession number can be found below: GitHub, https://github.com/Aczarnowska/MS-adverse-events-COVID19.

## Ethics Statement

The study was approved (approval No. 62/2021) by the Bioethics Committee at Collegium Medicum, Jan Kochanowski University in Kielce, Poland. Written informed consent for participation was not required for this study in accordance with the National Legislation and the Institutional Requirements.

## Author Contributions

ACz, JT, KK-T, AS, MA-S, HB-P, WB, and AK: conceptualization. ACz, OZ, MW, MM, KN, AJ-W, KR, BL, MP, IR-Ż, AP, MŚ-M, MSi, ACi, AW, AJ, MSta, KK, KD, AK-Ł, WG, AW-H, EK, JC-Ł, JU, AP-D, MC, AM, JKu, JKo, MB, MSto, KK-B, NN, PW, AP-P, MN, BZ-P, EJ, JZa, MM-J, JZw, BZ, and AP: patient enrollment and data collection. ACz and OZ: formal analysis. ACz, OZ, WB, and AK: methodology. AK: project administration. OZ: software. WB and AK: supervision. ACz: writing—original draft. WB and AK: writing—review & editing. All authors have read and agreed to the published version of the manuscript.

## Conflict of Interest

AK, WB, HB-P, AP-D, MA-S, EK, and KK received compensation for speaking and consulting services from Biogen, Bayer, Novartis, Roche, Merck, Teva, and Sanofi-Genzyme. MS received compensation for speaking from Roche, Novartis, Sanofi-Genzyme, and Biogen. MŚ-M received compensation for speaking and consulting services from Biogen, Novartis, Roche, Merck, and Sanofi-Genzyme. AJ received compensation for speaking services from Merck and SanofiGenzyme. MS received grant funding from Biogen and received compensation for speaking and consulting services from Biogen, Novartis, Roche, Merck, Sanofi-Genzyme, Bristol Myers Squibb, and Teva. AS, MM, KN, and MW received compensation for speaking and consulting services from Biogen, Bayer, Novartis, Roche, Merck, Teva, and Sanofi-Genzyme. They received also a grant from NCBIR (nr SZPITALE-JEDNOIMIENNE/18/2020). AK-L received grant funding from Novartis and received compensation for speaking and consulting services from Biogen, Bayer, Novartis, Roche, Merck, Teva, CSL Behring, Shire, and Sanofi-Genzyme. None of the agreements are relevant to the submitted work. The remaining authors declare that the research was conducted in the absence of any commercial or financial relationships that could be construed as a potential conflict of interest.

## Publisher's Note

All claims expressed in this article are solely those of the authors and do not necessarily represent those of their affiliated organizations, or those of the publisher, the editors and the reviewers. Any product that may be evaluated in this article, or claim that may be made by its manufacturer, is not guaranteed or endorsed by the publisher.
